# Intraoperative Neurophysiological Monitoring for Endoscopic Endonasal Approaches to the Skull Base: A Technical Guide

**DOI:** 10.1155/2016/1751245

**Published:** 2016-05-16

**Authors:** Harminder Singh, Richard W. Vogel, Robert M. Lober, Adam T. Doan, Craig I. Matsumoto, Tyler J. Kenning, James J. Evans

**Affiliations:** ^1^Stanford Hospitals and Clinics, Department of Neurosurgery, 300 Pasteur Drive, Stanford, CA 94305, USA; ^2^Safe Passage Neuromonitoring, 915 Broadway, Suite 1200, New York, NY 10010, USA; ^3^Sentient Medical Systems, 11011 McCormick Road, Suite 200, Hunt Valley, MD 21031, USA; ^4^Department of Neurosurgery, Albany Medical Center, Physicians Pavilion, First Floor, 47 New Scotland Avenue, MC 10, Albany, NY 12208, USA; ^5^Thomas Jefferson University Hospital, Department of Neurosurgery, 909 Walnut Street, Third Floor, Philadelphia, PA 19107, USA

## Abstract

Intraoperative neurophysiological monitoring during endoscopic, endonasal approaches to the skull base is both feasible and safe. Numerous reports have recently emerged from the literature evaluating the efficacy of different neuromonitoring tests during endonasal procedures, making them relatively well-studied. The authors report on a comprehensive, multimodality approach to monitoring the functional integrity of at risk nervous system structures, including the cerebral cortex, brainstem, cranial nerves, corticospinal tract, corticobulbar tract, and the thalamocortical somatosensory system during endonasal surgery of the skull base. The modalities employed include electroencephalography, somatosensory evoked potentials, free-running and electrically triggered electromyography, transcranial electric motor evoked potentials, and auditory evoked potentials. Methodological considerations as well as benefits and limitations are discussed. The authors argue that, while individual modalities have their limitations, multimodality neuromonitoring provides a real-time, comprehensive assessment of nervous system function and allows for safer, more aggressive management of skull base tumors via the endonasal route.

## 1. Introduction

For over a century in neurosurgery, the endonasal approach to the anterior skull base was recognized as a means to access sellar lesions [1–3]. First introduced by Schloffer in 1907, the reach of the endonasal approach greatly expanded with the introduction of numerous imaging technologies. For example, when Hardy introduced the intraoperative microscope in 1967, it revolutionized transsphenoidal surgery and improved its safety by combining improved magnification and illumination [4]. The most recent and, arguably, equally significant advancement in endonasal neurosurgery was the description of the use of the endoscope by Jankowski et al. in 1992 [5]. The subsequent rapid expansion of endoscopic intracranial surgery has permitted access to large areas of the cranial base and its associated pathology. Today, the reach of the endoscopic, endonasal approach (EEA) extends far beyond the sphenoid and sella to the entire ventral skull base via transcribiform, transplanum, transdorsum sellae, transclival, and transpterygopalatine fossa corridors [6–9]. Thus, multiple reports in the literature have demonstrated the utility of these approaches in reaching the anterior, middle, infratemporal, and posterior fossae.

Endoscopic endonasal surgery often requires working in close proximity to, and occasionally directly on, the critical neurovascular structures and cranial nerves that traverse the cranial base. Although the reported rates of vascular injury and cranial nerve deficits following endonasal cranial base surgery are low, these complications can have devastating effects. There is a relatively high potential for postoperative morbidity when addressing cranial base pathology, and all possible efforts must be made to limit that potential. Use of an endoscope clearly serves that goal by providing superior illumination and visualization when compared to the operating microscope. Any additional measures that improve the safety of endonasal surgery should be considered.

Intraoperative neurophysiological monitoring (IONM; neuromonitoring) can be used to further mitigate the risks associated with the EEA. IONM involves the use of physiological tests that can identify nervous system structures, as well as evaluating their functions, in real-time during surgery. IONM is based on the premise that the patient's neurophysiology changes in a measureable way, prior to the onset of permanent neurological deficit. Thus, critical, nontechnical changes in the neurophysiology data alert the neurosurgeon to perturbations in the nervous system which, if left unattended, could result in transient or permanent postoperative motor and/or sensory deficits. IONM endeavors not only to detect and identify iatrogenic nervous system dysfunction, but also to guide the use of surgical interventions and monitor their efficacy. Thoughtful data trend analysis, a keen understanding of neural elements at risk for injury, and knowledge of surgical technique (time-locking data changes to surgical/anesthetic conditions) are all critical in the neurophysiologist's assessment of these measures. In addition to monitoring the function of neural tissue, IONM is frequently used to identify and differentiate specific neural elements, such as motor cranial nerves, with the ultimate goal of preserving their baseline function through the duration of the surgical procedure.

Multimodality IONM has emerged as a standard approach to monitoring the nervous system under general anesthesia to improve safety and optimize surgical outcomes across a wide range of surgical procedures involving the central and peripheral nervous system. In the context of the EEA to the skull base, global cortical monitoring with electroencephalography, somatosensory evoked potentials, and transcranial, electrical motor evoked potentials is likely to be helpful in situations where the internal carotid arteries or their branches are at risk [10]. In the case of skull base approaches, this includes exposure of the parasellar region and cavernous sinus [11, 12]. Similarly, brainstem auditory evoked potentials (BAEP) are useful for detecting brainstem ischemia during surgery at or around the vertebrobasilar junction, as is the case for transclival approaches [12–14].

Intraoperative monitoring of the oculomotor, trochlear, and abducens nerves with needle electrodes placed in the inferior rectus, superior oblique, and lateral rectus muscles, respectively, has long been used during transcranial approaches to the cavernous sinus [15–17] and is equally applicable in many EEA procedures [18, 19], including cases in which the cavernous sinus is not accessed. The oculomotor nerve, for example, is vulnerable in the interpeduncular cistern via the transsphenoidal [20] and transplanum routes [21], with vascular compromise possible from injury to the inferolateral trunk of the cavernous carotid or its branches [20]. The trochlear nerve may be exposed at the ambiens division of the cisternal segment through the transellar transtubercular route, and ischemic injury may occur with injury to the superior cerebellar artery [22]. The abducens nerve, being the longest and most ventrally located cranial nerve at the level of the clivus and cavernous sinus, is particularly at risk during approaches to petroclival lesions via the midline transclival, paramedian suprapetrous, and medial petrous apex approaches [23]. In these cases, the risk may be increased by abnormal anatomy (e.g., medial displacement of the nerve by a petroclival tumor or upward displacement by a cisternal mass). Similar to the oculomotor nerve, it may also suffer vascular compromise by injury to the inferolateral trunk from the cavernous segment of the internal carotid artery. The trigeminal nerve, also at risk in some EEA procedures, may be violated in Meckel's cave via the transpterygoid corridor [24]. As EEAs are extended to the inferior clivus, as well as through the transcondylar, and transjugular corridors [25], attention must be placed on lower cranial nerve monitoring, including the glossopharyngeal, vagus, accessory, and hypoglossal nerves [26].

The techniques of multimodality monitoring, as they pertain to the EEA, are now relatively well-studied [11, 12, 14, 18, 19, 23, 26]. Each IONM modality has its benefits and limitations, and the power of neuromonitoring to detect or prevent iatrogenic injury emerges from the ability of the surgeon and neurophysiologist to combine these tests to monitor multiple neural structures and systems simultaneously. The risks of the surgical procedure guide the selection of IONM tests that form the multimodality monitoring plan for each individual patient. The surgical team is further empowered by the neurophysiologist's ability to quickly and accurately interpret and communicate the IONM data [27]. Here we report the IONM techniques that are available for the EEA. Each test modality is presented with a review of its utility in skull base surgery, recommendations for implementation, as well as benefits and limitations. We conclude with a discussion of how different IONM tests may be combined to optimize monitoring for different EEAs to the skull base.

## 2. Materials and Methods

IONM has been used during various surgical procedures for decades, gaining popularity in its modern form with the introduction of somatosensory evoked potentials for surgery in the 1970s [30, 31]. The ultimate goals of IONM are to reduce the risk of iatrogenic neural injury and to provide functional guidance to the surgical/anesthesia team, as necessary. In its evolution, IONM has expanded its scope and understanding, allowing it to be used effectively in myriad surgical procedures that include much of the central and peripheral nervous system. IONM is presently used in a wide range of intracranial procedures and has been shown to optimize surgical outcomes by significantly reducing the risk of iatrogenic injury to the nervous system. Indeed, iatrogenic injury is always one of the most feared complications of neurosurgery, and using the EEA to reach skull base pathology is no exception. Given the complex anatomy and physiology encountered in this approach, a multimodality monitoring plan is required to adequately assess the areas at risk and to increase the sensitivity and specificity of the totality of the monitoring plan. Depending on the location of the pathology, we commonly use electroencephalography (EEG), somatosensory evoked potentials (SSEPs), motor evoked potentials (MEPs), free-running and stimulus-triggered electromyography (EMG) of muscles innervated by cranial nerves, and brainstem auditory evoked potentials (BAEPs) to ensure full coverage of at risk neural structures (Table 1). Understanding the techniques for implementing these tests and knowing the advantages and drawbacks of each test, are necessary prerequisites for correct interpretation of response data.

### 2.1. Electroencephalography

Electroencephalography (EEG) began to be routinely used in the operating room in the 1970s to monitor cerebral perfusion in neurovascular procedures [32]. Today, EEG is often the standard of care at many institutions for both extracranial and intracranial vascular monitoring. While the intraoperative use of EEG recording has gained widespread popularity for vascular monitoring during carotid endarterectomy (CEA) [33–36] and cerebral aneurysm surgery [37, 38], it is not known whether or not EEG is routinely used in EEA to the cavernous sinus or parasellar regions where the internal carotid arteries and their branches are at risk. Preoperative clinical imaging helps to show the relationship between the lesion and the vascular anatomy, which is often enveloped or compressed by the tumor. The demonstrated utility of EEG as an intraoperative measure of cerebral blood flow [39] and the fact that it is a relatively noninvasive measure both make this IONM modality appropriate for EEA to the skull base.

**Figure 1:**
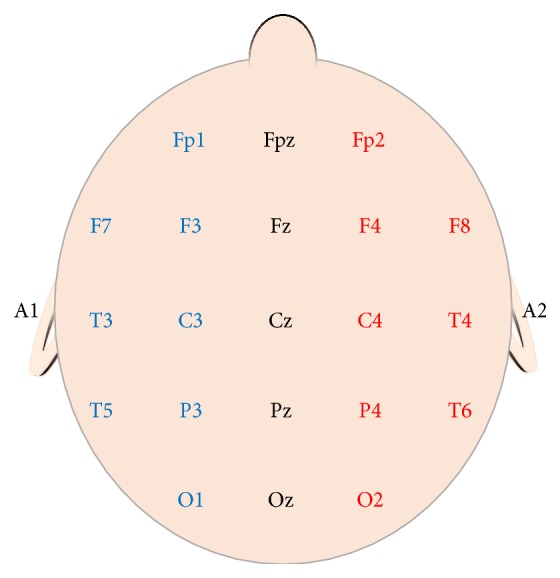
Common EEG recording locations using the International 10–20 System for Electrode Placement [28]. F: frontal; C: central; T: temporal; P: parietal; O: occipital; A: auricular; z: midline.

EEG is a free-running, real-time graphical representation of electrical potentials produced by neuronal activity in the cerebral cortex. EEG can be recorded using either subdermal needle electrodes or cup electrodes positioned on the surface of the scalp using the International 10–20 system for electrode placement (Figure 1) [28]. An 8–16 channel longitudinal bipolar montage can be assembled to adequately monitor gross cerebral cortical perfusion and with enough specificity to evaluate different aspects of the anterior and posterior circulation [37, 40]. Increasing the number of recording channels will help to increase sensitivity and specificity and can be considered in rare cases where a multimodal approach to neuromonitoring is not feasible. Extended 12 and 16 channel recording montages permit global cortical coverage of all 4 cerebral lobes across both hemispheres but may not be pragmatic in most cases. Depending on the vascular structures involved in the surgery, and whether anterior or posterior circulation is more at risk, the neurophysiologist will adopt a recording montage that evaluates relevant areas of cortex, maximizes sensitivity/specificity, and minimizes cognitive noise (i.e., excessive recording that provides no additional benefit to the analysis). As a matter of preference, we use 8–10 channel EEG recordings in the EEA, and this has been shown to provide 100% sensitivity and 100% specificity for detecting ischemia, at least during carotid endarterectomy surgery [41].

High pass filtering of ≤0.5 Hz and low pass filtering of 30 Hz will record the frequencies seen under general anesthesia; however, it is often the preference of the neurophysiologist to open the low pass filter to 70 Hz if an artifact-free EEG is recordable. Braiding or twisting the EEG wires will decrease electrical noise through facilitation of the amplifier's common mode rejection. High quality recordings are facilitated by maintenance of interhemispheric symmetry with regard to electrode placement and impedance (≤5 kΩ) between homo-topographic locations.

Raw (analog or digital) waveforms are monitored on all cases in which EEG is a selected IONM modality. Frequently the neurophysiologist will prefer to add quantitative EEG recordings, both numerical and graphical trend analyses using fast Fourier transformation (FFT), to facilitate the comparison of EEG activity across different time-points during surgery [37]. An EEG baseline can be established at the beginning of the procedure. If surface cup electrodes are used to record EEG, a baseline can be established prior to induction of anesthesia to identify any preexisting abnormalities in cerebral perfusion which may be evidenced by waveform asymmetry [37, 42].

Different anesthetic agents have widely different effects on EEG patterns [43]. Bolus administration of hypnotic agents, such as Propofol, can suppress EEG and preclude monitoring. Collaboration and communication between the neurophysiology and anesthesiology teams will help to ensure accurate interpretation of data. Indeed, an important benefit of multimodality monitoring is that anesthesia-induced changes in EEG patterns help the neurophysiologist to predict and accurately interpret changes in other IONM modalities, thereby avoiding false positive findings. The experienced neurophysiologist will use EEG recordings to assist the anesthesiologist with maintaining an appropriate level of sedation. EEG recordings do not replace monitoring of clinical parameters such as blood pressure, heart rate, gas concentrations (O_2_/CO_2_), and peak pressures; rather, EEG may add valuable, complimentary information about global cortical activity [44]. As a basic measure of arousal, many anesthesiologists also employ a “consciousness monitor,” which consists of 2–4 electrodes placed on the patient's forehead. The monitor records a combination of EEG and EMG (bispectral) and uses an algorithm to calculate an index value between 0 and 100, which has an inverse relationship to the “depth” of anesthesia. The utility of the bispectral index (BIS) to reduce the incidence of intraoperative awareness is a matter of debate [45–47], and the integration of EEG and EMG into a single index has led to false interpretation [48]. Using multimodality monitoring, the neurophysiologist has the capability to analyze muscle tone and cortical activity separately using EMG and EEG, respectively. This provides the anesthesiologist with the ability to target drug treatment. Thus, increased muscle tone (*patient is reactive*) can be resolved with administration of narcotics (e.g., fentanyl), and increased cortical activity (*patient is light*) can be resolved with administration of hypnotics (e.g., Propofol). While the anesthesiologist may wish to rely on the use of a BIS monitor, this may not always be possible in endoscopic endonasal surgery. Specifically, in cases where a navigation-registration mask is used, insufficient space would be left on the forehead for anesthesia to place the monitor. In this circumstance, the neurophysiologist can use EEG and EMG to provide the anesthesia team with valuable information that will complement monitoring of patient vitals and other clinical variables.

Although it is rare, iatrogenic vascular injury does occur and can have devastating results. When this occurs, EEG can be used noninvasively to monitor and predict significance in a real-time fashion, without substantial temporal delay. Studies of analog EEG have given rise to a multitude of threshold criteria for identification of hypoperfusion. Generally, decreased cerebral blood flow causes suppression of EEG amplitude and slowing [38]. While there are no studies specific to the EEA, thresholds used to detect cerebral ischemia in cerebral aneurysm and carotid endarterectomy surgery may be applied. A reasonable starting point is to use criteria of >50% loss of overall EEG amplitude or fast activity, or >50% increase in slow activity [38]. These measures can be used to help determine the need for the patient to be taken to interventional radiology for exploration and treatment and can also help to show evidence of vasospasm, which otherwise may be undetectable. In the event of vascular rupture, EEG can also be used to assess the amount of pressure applied by packing the surgical site, ensuring that adequate cortical perfusion is maintained. While EEG is useful for measuring adequacy of cortical perfusion, the surgeon must be aware of its limitations. In particular, EEG is not an effective method for monitoring subcortical perfusion or the functional status of eloquent cortex. These structures and functions must be assessed by other measures, and this underscores the importance of multimodality neuromonitoring.

### 2.2. Somatosensory Evoked Potentials

Somatosensory evoked potentials (SSEPs) are one of the most commonly used IONM modalities and can be used to assess many different aspects of both the peripheral and central nervous system. Their use in neurovascular procedures is well documented, and there are numerous reports documenting their use during skull base procedures, in particular [11, 49, 50]. SSEPs provide valuable information on the status of both cortical and subcortical function, both in regard to perfusion, as well as long-tract neural integrity. This adds needed information to the clinical picture painted by EEG, which reflects only cortical function, and increases the sensitivity and specificity of the monitoring plan [51, 52].

SSEPs are recorded using electrodes placed on the scalp following electrical stimulation of peripheral nerves on all 4 extremities, including bilateral ulnar or median nerves at the wrists, and posterior tibial nerves at the ankles. For median nervestimulation, the cathode is placed between the tendons of the palmaris longus and the flexor carpi radialis muscles, 2 cm proximal to the wrist crease. The anode is placed 2-3 cm distal to the cathode or on the dorsal surface of the wrist. For ulnar nerve stimulation, the cathode is placed between the tendons of the flexor digitorum superficialis and the flexor carpi ulnaris muscles, 2 cm proximal to the wrist crease. The anode is placed 2-3 cm distal to the cathode or on the dorsal surface of the wrist. For posterior tibial nerve stimulation, the cathode is placed over the posterior portion of the medial surface of the ankle, 1-2 cm distal and posterior to the medial malleolus. The anode is placed 2-3 cm distal to the cathode. Alternate stimulation sites or alternate peripheral nerves can be used when comorbidities preclude recording from these preferred sites.

Peripheral nerve stimulation is commonly achieved with the use of stick-on surface electrodes or subdermal needle electrodes. The authors prefer the latter, particularly for long-duration procedures in which the adhesive from surface electrodes may degrade and cause stimulation failure and/or stimulus shunting due to the development of a salt bridge.

Interleaving square wave pulses of 200–400 *μ*sec duration are used at a frequency of 2–5 Hz and using a supramaximal stimulation intensity. This intensity is 20% above the threshold for muscle twitch in the distal muscles innervated by the stimulated nerve. While each patient requires slightly different stimulation parameters to optimize SSEP data, the authors recommend a pulse duration of 300 *μ*sec, a frequency of 4.7 Hz, and an intensity of 25–45 mA for the ulnar/median nerves or 35–50 mA for the posterior tibial nerves.

**Figure 2:**
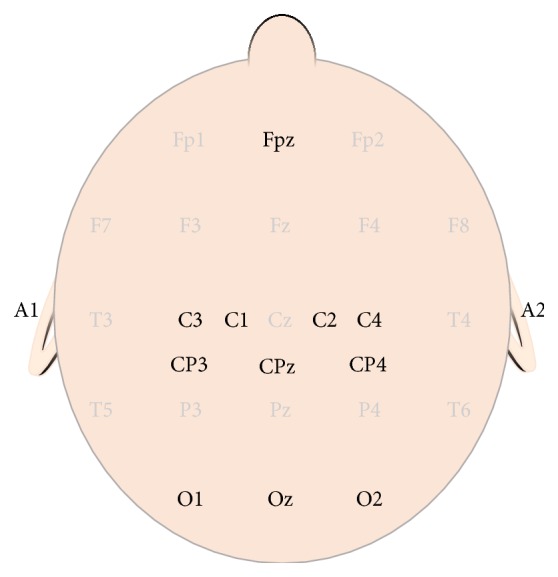
Electrode positions used for stimulating tceMEP, and recording SSEP, BAEP, and VEP. All recording locations are based on the International 10–20 System for Electrode Placement [28]. F: frontal; C: central; CP: midway between central and parietal; O: occipital; A: auricular; z: midline; Cs2: cervical spine (not shown).

SSEPs can be recorded using either subdermal needle electrodes, or cup electrodes positioned on the surface of the scalp using locations modified from the International 10–20 system for electrode placement (Figure 2). Following ulnar or median nerve stimulation, subcortical SSEPs are recorded with a latency of 13 msec (N13) over the 2nd cervical vertebra (Cs2), and cortical SSEPs are recorded with a latency of 20 msec (N20) from the contralateral cerebral hemisphere (CP3 or CP4). All recording sites are referenced to Fpz. Following posterior tibial nerve stimulation, subcortical SSEPs are recorded with a latency of 29 msec (N29) over the 2nd cervical vertebra (Cs2), and cortical SSEPs are recorded with a latency of 37 msec (P37) from the cerebral vertex (CPz). The cortical SSEP may also be recorded from the ipsilateral cerebral hemisphere (CP3 or CP4) due to paradoxical lateralization. A transcortical montage (CP3-CP4, CP4-CP3) can often be used to facilitate signal acquisition if initial cortical amplitudes are low.

Bandpass filters of 30–500 Hz are used for subcortical recordings, while 30–300 Hz is typically optimal for cortical recordings. Peripheral recording sites, such as Erb's point or popliteal fossa, are used by some labs to assist with technical troubleshooting, but the authors have not found these methods to be of sufficient benefit as to warrant inclusion in our monitoring plan. If the reader opts to employ peripheral recordings, we recommend bandpass filters of 30–1500 Hz. A recording epoch of at least 50 msec is recommended for upper extremity SSEPs, and 100 msec for lower extremity SSEPs. The SSEP is an averaged response, which can take dozens to hundreds of trials to fully resolve, ranging in time from 30 seconds to 2 minutes, depending on the amount of unresolved electrical noise in the environment. Contemporary IONM systems usually permit full resolution of an SSEP waveform in under 30 seconds, which is a significant improvement over decades past. Braiding or twisting the SSEP recording wires will decrease electrical noise through facilitation of the amplifier's common mode rejection. High quality recordings are facilitated by maintenance of interhemispheric symmetry with regard to electrode placement and impedance (≤5 kΩ) for all recording locations.

SSEP baselines should be recorded after induction, but prior to any significant patient positioning. This will help to detect and correct pressure or traction on the brachial plexus or peripheral nervous system. An alarm criterion of a 50% amplitude decrease and/or a 10% latency increase are traditionally used, both for positioning issues and for true iatrogenic changes. While SSEPs provide information regarding the functional status of eloquent cortex and patient positioning, they still have several limitations. For example, these long-tract sensory pathways are not fully sensitive to subcortical ischemia [53] and do not provide any information specific to the motor pathways. In cases where the ischemic penumbra is small or slow to develop, or in cases where only the motor pathways are affected, SSEPs may remain unchanged from baseline parameters, despite evolving hemiparesis [54]. These limitations can lead to false negative neurophysiologic findings and may need to be supplemented by additional modalities, such as EEG and motor evoked potentials.

### 2.3. Motor Evoked Potentials

Transcranial electrical motor evoked potentials (tceMEPs) have played a role in the operating room since it was first demonstrated that the pulse-train stimulation technique could successfully evoke MEPs in the anesthetized patient [31, 55]. While routine use of tceMEP monitoring began in spinal surgery in the 1980s, it is now also commonly used in many supratentorial [56–61] and infratentorial [62–66] procedures, as well as procedures in which peripheral nerves or spinal nerve roots are at risk [67–69]. Given the limitations of EEG and SSEPs mentioned above, the addition of tceMEPs to the multimodality IONM plan can paint a more comprehensive picture of nervous system function when monitoring cases using the EEA. Inclusion of tceMEPs monitoring is the only means of detecting insult to the long-tract motor pathways. Although the utility of tceMEPs in detecting functional motor change has been demonstrated in a wide range of intracranial procedures [56–66], their efficacy using the EEA is scarce.

Motor evoked potentials are recorded from skeletal musculature following electrical stimulation of primary motor cortex via electrodes placed over C1-C2 or C3-C4 cranially (Figure 2). Corkscrew or subdermal needle electrodes are commonly used. Anodal stimulation works best to activate the corticospinal tract and elicit MEPs from upper and lower extremity muscles contralateral to the stimulated cerebral hemisphere. A multipulsed train of square wave constant voltage stimuli of 50 to 75 *μ*sec duration is used. The pulse-train stimulation method is required to overcome the effects of anesthesia. The amount of voltage required to elicit MEPs can vary significantly between patients. The number of pulses can range from 3 to 9 and the interstimulus interval (ISI) can range from 1 to 4 msec (equal to a frequency of 1000–250 Hz, resp.). All stimulation parameters, including voltage, are tailored to the individual patient to optimize data and minimize the possibility of false positive or false negative findings.

Motor evoked potentials are recorded using bipolar subdermal needle electrodes placed in upper and lower extremity muscles bilaterally. For example, the neurophysiologist may elect to record from the extensor carpi radialis, first dorsal interosseous, tibialis anterior, and abductor halluces muscles. Many alternate recording sites are available. Use of multiple recording sites ensures adequate coverage of long-tract corticospinal function and has the added benefit of helping to detect and correct positional changes. A recording epoch of at least 100 msec is required and bandpass filters of 10–3000 Hz are recommended. The tceMEP is not an averaged response; thus, each test gives immediate feedback regarding corticospinal tract function. Stimulation may cause slight patient movement, often requiring a brief surgical pause for testing. This can be done in the seconds during which surgical instruments are exchanged. The surgeon must remain aware that tceMEP testing is not performed continuously during surgery.

One concern regarding tceMEP monitoring during intracranial procedures is the risk of false negative findings due to excessive stimulation. To mitigate this risk, the voltage and all stimulating parameters are kept purposefully low to help limit electrical spread to the cortical layers of the stimulated cerebral hemisphere. Stimulation parameters that are set too high will bypass the cerebral cortex and depolarize the pyramidal tracts at subcortical levels, potentially leading to false negative recordings in the event of eloquent motor cortex ischemia. While this is not of concern in spinal procedures, it should always be considered in cranial cases. Including contralateral myotomes in the ipsilateral recording trace window helps to identify this limitation. For example, anodal stimulation of the right cerebral hemisphere will produce muscle recordings on the left side of the body. In the recording trace window for the left side of the body, one can include right side myotomes as well. Responses generated from the left myotomes, with absence of responses from the right myotomes, can help to demonstrate focal stimulation that is confined to the right cortical hemisphere. These techniques can help to predict and prevent those rare occasions when other monitoring modalities, such as SSEP or EEG, do not display signal change in the face of evolving hemiparesis.

When the EEA to the skull base poses risk to the brainstem via the transclival approach, for example, the medullary pyramids are at risk and tceMEPs provide the added benefit of monitoring long-tract motor function. While corticospinal tract monitoring has been increasingly utilized across various neurosurgical procedures, stimulation of the corticobulbar tract for monitoring cranial nerve motor evoked potentials (CrN MEPs) has not gained the same popularity. As previously mentioned, lesions in the cavernous sinus or around the clivus will frequently compress or surround the cranial nerves. Although the use of electrically triggered EMG (see Section 2.4) is the gold-standard for cranial nerve IONM, it is often the case that large tumors must be partially debulked prior to localization of neural elements. This initial debulking of the tumor can result in iatrogenic injury, prior to the baseline stimulation. CrN MEPs allow one to establish a baseline response prior to surgical manipulation, much in the same way where upper and lower extremity baselines are established. CrN MEP testing also permits assessment to occur on a more frequent basis, as it is much faster than pausing the surgery to stimulate with a handheld probe.

CrN MEPs are recorded from cranial-nerve-innervated muscles following transcranial electrical stimulation of primary motor cortex. Dong and colleagues [70] first described the technique for eliciting CrN MEPs from muscles innervated by the facial nerve, and the utility of this technique has since been investigated for various other cranial nerves [71–73]. In order to limit electrical stimulation to the most lateral aspects of the motor homunculus, a hemispheric stimulation montage is used. The anode is placed contralateral to the operative side (C3 or C4; Figure 2), and the cathode is paced at the vertex (Cz).

Stimulus parameters are similar to those listed above for corticospinal tract monitoring. A multipulsed train stimulus is required to overcome the effects of anesthesia. It is imperative to limit electrical stimulation to the poly-neuronal corticobulbar tract and not allow electrical spread through deeper structures or around the periphery of the scalp and face. Any response that is recorded following a single-pulse stimulus is likely generated by activation of the peripheral pathways. Failure to limit spread can bypass the corticobulbar pathway and cause direct activation of cranial nerves, which can result in false negative findings. The idea that CrN MEP latency is a good predictor of whether or not the response is generated centrally or peripherally has recently been challenged [74, 75].

CrN MEPs are recorded using the same electrodes that are used for EMG recordings (see Section 2.4). Frequently, there is also a great deal of stimulus artifact present in the responses, which can obscure CrN MEPs due to their inherent short latency. The hemispheric stimulation montage produces reliable responses and helps to eliminate the stimulus artifact that is common to CrN MEPs. Additionally, we have found that raising the high pass filter from 10 to 100 Hz can reduce stimulation artifact.

Interpretation of tceMEPs is complicated by their inherent trial-to-trial amplitude variability and the lack of consensus in the literature regarding criteria for alert. In supratentorial and brainstem surgery, major alert criteria for tceMEPs include disappearance or consistent >50% amplitude reduction [57, 58, 65, 76–78]. While the same alert criteria are commonly used for CrN MEP interpretation [70, 79–82], the situation is further clouded by the observation that intraoperative test results do not always correlate well with the postoperative neurological outcome [10, 80, 83]. Due to the potential for a high number of false positives and negatives, CrN MEPs have not become routine. Further investigation is required before predictive values can be established that allow CrN MEPs to be used during skull base endoscopic procedures. Thus, while tceMEPs are frequently utilized to monitor eloquent cortical and long-tract motor functions, reliable monitoring of cranial nerve motor function requires other methods, such as free-running and stimulus-triggered electromyography.

### 2.4. Electromyography

Electromyography (EMG) is recorded in surgery to monitor somatic efferent nerve activity and assess the functional integrity of individual nerves. First introduced in the 1960s as a means to assess facial nerve function during exploratory parotid surgery [84, 85], EMG recording techniques were later adapted for intracranial [86], spinal [87], and peripheral nerve surgeries [88]. A large volume of literature devoted to EMG use during intracranial surgery is devoted to facial nerve monitoring in the cerebellopontine angle [89, 90], and there is a growing number of reports on the use of this technique with the EEA to the skull base [18, 19, 26]. With this approach, EMG recordings are particularly important to identify cranial nerves and guide tumor resection when the pathology involves the cavernous sinus or retroclival regions. While other techniques used in multimodality IONM provide valuable information about nervous system function, EMG is the only method that can (1) provide real-time feedback about neural activation throughout the course of surgery, (2) accurately detect and localize motor or mixed motor-sensory nerves embedded within tumor, and (3) reliably assess the integrity of cranial nerve motor functions before, during and after tumor resection.

The basic premise of EMG is that depolarization of a motor nerve produces a recordable electrical potential within one or more muscle(s) innervated by that particular nerve. This activity is recorded using subdermal or intramuscular needle electrodes, unless otherwise noted below. A bipolar montage is frequently employed, consisting of two active recording leads placed within the same muscle. Alternatively, a referential montage may be used in which a single electrode is placed in the muscle of interest, and a reference electrode is placed in a neutral location.

For each motor or mixed sensory/motor cranial nerve, the following muscles are selected: inferior rectus or superior rectus (CN III); superior oblique (CN IV); masseter or temporalis (CN V); lateral rectus (CN VI); orbicularis oculi, orbicularis oris and mentalis (CN VII); stylopharyngeus (CN IX); vocalis (CN X); upper trapezius (CN XI); and tongue (CN XII). Recording from extraocular muscles for monitoring CNs III, IV, and VI requires knowledge of ocular anatomy and associated vasculature. Careful electrode placement will ensure the integrity of the sclera. Several recording methods have been reviewed elsewhere [91]. We use a commercially available subdermal needle electrode which is prebent to approximately 90°. With the eye in the closed position, the neurophysiologist inserts the needle into the extraocular muscle of interest and carefully advances the electrode along the bony ridge of the orbit until it is fully inserted. A small piece of Transpore*™* tape is used to secure each electrode as the others are placed. One electrode is placed for each of the extraocular muscle monitored, and each recording is referenced to a needle inserted into the frontalis muscle of the forehead. Tegaderm*™* film is applied to protect the eyes during surgery. When the glossopharyngeal nerve is monitored, prebent subdermal needle electrodes are placed in the soft palate with the aid of a curved hemostat (although the utility of glossopharyngeal nerve monitoring has recently been challenged) [92]. A commercially available endotracheal tube with surface-mounted electrode contact can be used to monitor the vagus nerve. For all other cranial-nerve-innervated muscles, pairs of straight, subdermal needle electrodes are used for recording EMG. If a mask registration device is used for neuronavigation, then all EMG electrodes must be placed after registration is complete.

Bandpass filters of 10–3000 Hz will capture all frequency components of EMG. The low pass filter can be reduced to parry excessive high pass noise. A vertical screen resolution of 50–200 *μ*V/division is appropriate to visualize spontaneous and evoked EMG activity. Accurate interpretation of EMG is facilitated by simultaneous visual and auditory monitoring, so a speaker is used in parallel to provide concurrent auditory feedback. Whereas incidental motor cranial nerve activity is monitored with a free-running, spontaneous EMG recording (S-EMG), stimulus-triggered EMG (T-EMG) recordings are time-locked to delivery of an electrical pulse and used to map the location of cranial motor nerves, as well as assessing their functional status, as described below.

Free-running S-EMG is recorded throughout the course of the surgery and it provides real-time feedback whenever a nerve is activated. A recording time base of 200–1000 msec/division is used. Several distinct firing patterns of S-EMG activity may be recorded [29, 93], and the surgeon should be able to identify them by sound [94]. While the sound of EMG activity may serve to heighten the surgeon's awareness, not all patterns of EMG activity are cause for concern and attempting to differentiate EMG patterns by sound alone is inadvisable. Also, in the electrically hostile environment of the operating room, one must be able to detect true neuronal activity and distinguish it from 60-cycle noise and other forms of electrical interference, which should be reduced or eliminated when possible. This underscores the importance of having an experienced neurophysiologist present to interpret the waveforms.

In general, the surgeon should be familiar with two patterns of EMG activity:* neurotonic* and* motor unit* discharges.* Neurotonic* discharges are characterized by irregular, high frequency (50–300 Hz) burst and train EMG.* Motor unit* discharges are characterized by relatively regular and sustained low-frequency EMG. Of greatest concern is* neurotonic* EMG, which is caused by nerve compression, traction, or blunt trauma.* Motor unit* potentials are volitional, secondary to increased muscle tone, and can be informative about insufficient patient sedation (*patient is reactive*). This is frequently resolved with administration of narcotic agents, such as fentanyl.

**Figure 3:**
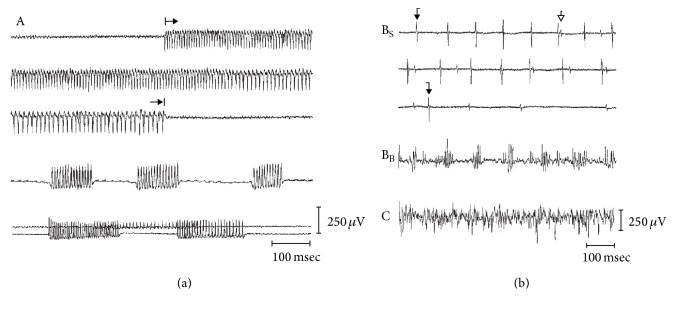
Electromyographic train activity. (a) Examples of A-trains of various duration and frequency. (b) Waveforms defined as B-trains with spikes (B_S_), and B-trains with bursts (B_B_) as predominant single components. The lowest tracing represents irregular EMG activity, called a C-train. C-trains are frequently recorded from laryngeal muscles at rest. The presence elsewhere is evidence of muscle tension and suggest insufficient sedation. Of these different forms of S-EMG activity, only A-trains are associated with neural injury. Figure from Romstöck et al. [29], with permission.

Figure 3 depicts various firing patterns that one my encounter when recording EMG from muscles innervated by cranial nerves. When EMG firing patterns are classified based on waveform morphology, amplitude, frequency, and duration, most patterns of EMG activity are benign in terms of predicting postoperative morbidity, and only “A-train” activity is highly predictive of postoperative nerve dysfunction [29]. A-train activity is a form of* neurotonic* EMG activity characterized by a sudden onset, irregular, high amplitude (100–200 *μ*V), and high frequency (60–210 Hz) discharge that can last for several seconds. Trains lasting longer than 10 seconds have been associated with postoperative deficit [95]; however, it is critically important to remain aware that absence of S-EMG activity is not necessarily indicative of stable nerve function. Indeed, it has been shown that EMG activity may be absent following serious nerve injury, including sharp dissection [96, 97]. Thus, the surgeon must remain aware that free-running s-EMG has limited sensitivity to nerve injury. Nevertheless, recording S-EMG is particularly important during tissue retraction and tumor dissection, and real-time auditory feedback can serve as a valuable asset to the surgeon when* neurotonic* EMG activity is recorded. Any observation of* neurotonic* EMG activity serves as a criterion for alarm and a surgical pause should be initiated immediately to identify and address the problem.

T-EMG is recorded at specific points in surgery, as opposed to throughout. A hand-held probe, insulated to the tip, is used to deliver 50–100 *μ*sec, 2.1 Hz constant current stimulation [98]. When a nerve is depolarized, T-EMG is recorded in the form of compound muscle action potentials (CMAPs). The recording window is time-locked to the onset of the stimulus, allowing the latency and amplitude of CMAPs to be quantified and compared.

The T-EMG recording epoch can range from 20 to 50 msec (2–5 msec/division), depending on the expected latency of the CMAP. The window sensitivity is initially set to 50 *μ*V/division but may be increased to quantify high amplitude CMAPs. Signal gain and bandpass filters are the same as S-EMG. Whenever T-EMG is employed, it is essential to eliminate fluids in the field by applying suction during stimulation. This helps to avoid current shunting, in which a low-resistance path (fluid) directs electrical stimulation away from the desired target (nerve). This can cause a false positive result (i.e., CMAP recorded from unexpected location due to unintended depolarization of a different nerve), or a false negative result (i.e., no CMAP recorded because current bypasses the target nerve and flow directly to the return electrode). When T-EMG is employed, monopolar and bipolar stimulating techniques are available, depending on the needs of the surgeon.

Monopolar stimulation allows current to spread through tissue, utilizing a return electrode placed somewhere on the patient's body outside of the surgical field, such as the shoulder or sternum. This technique is optimal for probing nonneural tissue (i.e., tumor, muscle, and bone) to detect underlying neural elements and rule out their presence. The neurophysiologist is informed about the identity and location of nerves by the threshold, latency, and amplitude of the CMAP, as well as the muscle(s) from which it is recorded. Ergo: the closer the neural element is to the locus of stimulation, the lower the threshold required to elicit a CMAP. Additionally, as the locus of stimulation approaches the nerve, the CMAP is likely to exhibit a shorter latency and higher amplitude, and with less spread of excitation to nearby nerves. As a general rule, when a CMAP is evoked with 1.0 mA or less, this is evidence of neural proximity and the surgeon should dissect with caution. More distal cranial nerves may require higher levels of stimulation for depolarization, and the CMAP may exhibit a smaller amplitude and longer latency. Whenever the expected result of stimulation is absence of a response, it is advisable to use a positive control to demonstrate efficacy of stimulation. This can be accomplished either by increasing the current until a CMAP is recorded, or by stimulating an exposed motor/mixed nerve and recording the CMAP. While direct nerve stimulation is always preferred prior to tumor resection, it is often the case that tumors are of sufficient size and they must partially be debulked prior to exposure of neural elements. Monopolar stimulation is advantageous in this situation because ruling out the presence of underlying motor/mixed cranial nerves permits rapid, safe extraction of nonneural tissue.

Bipolar stimulation limits the spread of current through tissue, because the active and return electrodes are very close together, usually <3 mm apart. This technique is preferred when the surgeon endeavors to identify a nerve or determine whether or not a nerve is functional. Identification of a nerve is accomplished with direct electrical stimulation, and the muscle(s) from which the CMAP is recorded help to reveal the identity of the nerve. For example, if an unidentified nerve is stimulated and CMAPs are recorded from the lateral rectus muscle, then the nerve in question is the abducens nerve (CN VI). Basic motor/mixed nerve functionality is assessed by establishing a CMAP* threshold*, which is defined as the minimum current (mA) required to evoke a CMAP. Beginning at 0.00 mA, the current is carefully increased by 0.01 mA increments until a CMAP is recorded with minimal spread to other nerves/muscles. Nerves should be stimulated frequently during tumor debulking to assess changes in threshold. At the end of the procedure, the nerve should be stimulated on each side of the tumor, proximal and distal to the brainstem, with little expected variation in the CMAP threshold. In the interest of prognostication, a number of methods have been reported for evaluating facial nerve function [99–105], but it is unclear if these methods can be generalized to address all motor or mixed sensory/motor cranial nerves.

### 2.5. Brainstem Auditory Evoked Potentials

The auditory brainstem response (ABR), also known as the brainstem auditory evoked potential (BAEP), is recorded in surgery to monitor vascular perfusion, as well as functional integrity, of the ascending auditory system, beginning with the vestibulocochlear nerve (CN VIII) and including associated brainstem tracts and nuclei up to the inferior colliculus [13, 53, 106–108]. Inclusion of BAEPs into the multimodality IONM plan is recommended whenever there is potential for brainstem ischemia in surgery. Inclusion of BAEPs in the multimodality IONM plan is standard for monitoring brainstem perfusion during open posterior fossa surgery [13]. As the EEA has expanded to reach pathology beyond the clivus and to the foramen magnum, inclusion of BAEPs is recommend to compliment other monitoring modalities [12, 14].

BAEPs are recorded in response to auditory (click) stimulation delivered to the ears. The stimulus is delivered through expanding foam earbuds placed in the external auditory canal. The click consists of a 99 dB (nHL), 100 *μ*sec pulse presented to each ear in interleaving fashion at a frequency range of 9.1–17.1 Hz. The polarity of the click may be rarefaction, condensation, or both (alternating). BAEPs can be recorded using either subdermal needle electrodes or cup electrodes positioned on the surface of the scalp using locations modified from the International 10–20 system for electrode placement (Figure 2). A referential recording montage is used in which active recording electrodes are placed at A1 or A2, and recordings are referenced to Cz.

Bandpass filters of 100–1500 Hz are common, and a recording epoch of at 15–20 msec is recommended. Because the BAEP is a far-field response, it is small in amplitude (usually less than 1 *μ*V), but robust and repeatable when hearing is intact. The BAEP is an averaged response, which can take hundreds to thousands of trials to fully resolve, ranging in time from 1 to 3 minutes, depending on the amount of unresolved electrical noise in the environment. Contemporary IONM systems usually permit full resolution of BAEP waveform in under 1 minute, which is a significant improvement over decades past. Braiding or twisting the BAEP recording wires will decrease electrical noise through facilitation of the amplifier's common mode rejection. High quality recordings are facilitated by maintenance of interhemispheric symmetry with regard to electrode impedance (≤5 kΩ) for all recording locations.

The BAEP consists of a waveform with approximately 5 distinct peaks, labeled (I)–(V), which reflect neuronal activity through the ascending auditory pathway. The neural generators for the peaks are (I) distal auditory nerve, (II) proximal auditory nerve, (III) cochlear nucleus, (IV) superior olivary complex, and (V) lateral lemniscus or inferior colliculus [109]. There are several longer latency (nonbrainstem-generated) peaks that represent higher thalamic and cortical auditory processing, but these peaks are suppressed by anesthetic agents [110]. The technical and pathological mechanisms that may underlie changes in the BAEP are numerous [109].

BAEP baselines should be established before traversing the clivus as the petroclival approach places the brainstem at risk for ischemia secondary to vascular compromise (arterial compression, rupture, or vasospasm). Alarm criteria are defined as persistent decreases in amplitude of greater than 50% of wave (V) and/or persistent absolute latency increase of the peak of wave (V) which equals or exceeds 0.5 milliseconds [12]. Knowledge of the neural generators for each wave can reveal the location and extent of the injury. For example, a major ischemic accident secondary to basilar artery rupture may result in disappearance of waves (I)–(V) because perfusion of the cochlea via the internal auditory artery may be compromised. Ischemia of higher brainstem structures may preserve waves (I)–(III) but abolish or delay wave (V). When these changes do not resolve during the course of surgery, postoperative deficits are to be expected [109]. A more comprehensive review of mechanisms underlying changes in the AEP is beyond the scope of this paper.

### 2.6. Visual Evoked Potentials (VEP)

Visual Evoked Potentials (VEPs) are recorded in surgery to monitor the visual pathway, beginning with the prechiasmatic optic nerve and ending with the striate cortex. Surgical approaches to the parasellar region of the anterior skull base pose risk to the optic nerve and iatrogenic visual field deficits are a serious concern. Since the introduction of VEPs as an intraoperative monitoring modality [111], their questionable prognostic value has been widely published [112–115]. Nevertheless, the numerous reports of VEP monitoring during the EEA to the anterior skull base warrant their inclusion in this paper [111–114, 116–126].

Goggles or contact lenses can deliver a flash stimulus using light-emitting diodes (LEDs) to the anesthetized patient. The flash stimulus is presented for 200–400 msec, and a typical stimulation frequency can range from <1 Hz to 3 Hz. VEPs can be recorded using either subdermal needle electrodes or cup electrodes positioned on the surface of the scalp using locations modified from the International 10–20 system for electrode placement (Figure 2). A referential recording montage is used in which active recording electrodes are placed at O1, O2, and Oz, with references either to A1/A2.

Bandpass filters of 1 to 300 Hz are common and can be narrowed if stimulation artifact is encountered. A recording epoch of at least 200 msec is required. The VEP is an averaged response, which can take dozens to hundreds of trials to fully resolve, ranging in time from seconds to minutes, depending on the amount of unresolved electrical noise in the environment. Similar to other averaged evoked potentials, braiding or twisting the recording wires will decrease electrical noise, and high quality recordings are facilitated by maintenance of interhemispheric symmetry with regard to electrode impedance (≤5 kΩ) for all recording locations.

The VEP is usually recorded at its first negative deflection with a latency of approximately 70 msec (N70), followed by its first positive deflection with a latency of approximately 100 msec (P100). Alert criteria include (1) total loss of waveform, (2) loss of peak in the waveform, (3) latency increase >2 standard deviations from baseline, or (4) amplitude decrease >50% from baseline [112].

Many lesions arising in the parasellar region result in compression/encasement of the optic nerve or chiasm, often resulting in preoperative clinical visual disturbances. It has been demonstrated that decompression of these visual pathways can actually improve postoperative visual neurological testing [127]. Additionally, with the ability to detect evolving injury to the healthy, unimpaired optic tract is appealing. Unfortunately, there is little in the way of evidence that intraoperative VEPs can accurately detect and help to prevent iatrogenic injury to the visual pathways. In one study of VEP monitoring during transnasal surgery of 22 patients, intraoperative latency or amplitude change did not correlate with immediate postoperative improvement or deterioration [117]. A larger, more recent study of VEP monitoring during transnasal surgery in 53 patients demonstrated no association between VEP waveforms and postoperative visual outcomes [128]. Lack of prognostic value is common with VEPs in surgery. Indeed, the biggest limitations for the use of VEPs to prevent postoperative visual field deficits include variability of the response secondary to anesthetic regimen and stimulus delivery [115]. Owing to the inconsistency of these recordings, which leads to both false positive and false negative findings, VEPs are not recommended during the EEA to the skull base.

### 2.7. Anesthesia

Every anesthetic agent administered during surgery will affect neurophysiologic recordings to varying degrees; VEPs are particularly affected, whereas BAEPs show little fluctuation despite the anesthetic regimen. The success of the monitoring plan relies on cooperation and continuous communication with the anesthesia team. We recommend using total intravenous anesthesia (TIVA; no inhalational anesthetic agents) to facilitate IONM during this surgical approach. TIVA helps to reduce the dose-dependent attenuation of signal amplitudes that are usually seen when using inhalational agents, and helps to improve the signal-to-noise ratio, thereby optimizing the monitoring plan [34, 129, 130].

As it relates to tceMEPs, the common agents used in TIVA produce less inhibition at the pyramidal tract synapse on *α*-motor neurons of the spinal cord [131–137]. This allows the multipulse descending summation to overcome the effects of anesthesia more readily, ultimately producing recordable CMAPs. The patient must also be sufficiently free of pharmacological blockade of the neuromuscular junction to allow both tceMEPs and EMG to be sensitively recorded. Absence of neuromuscular blockade or sufficient clearance/reversal for reliable monitoring can be documented by using “train-of-four” (TOF) monitoring, which records muscle twitches in response to stimulation of a peripheral nerve [138]. Using supramaximal stimulation at 2.0 Hz, TOF can be recorded from the distal extremities to stimulation of the ulnar and posterior tibial nerves, and responses can be recorded from the first dorsal interosseous and abductor halluces muscles, respectively. Short-acting neuromuscular blocking agents can be given to facilitate endotracheal intubation but are then discontinued for the remainder of the procedure. Currently, there is literature to support partial neuromuscular blockade to help to limit patient movement during surgery where IONM is occurring; however, partial blockade affects different muscle groups to varying degrees [139, 140] and can be especially variable in a patient with preexisting neurological dysfunction [130, 138].

This regimen of anesthesia (TIVA and a full TOF) requires diligent teamwork between the neurophysiologist and the anesthesia team to ensure that there is no patient movement and that analgesia is adequately controlled. The neurophysiologist, having means to assess the patient's level of sedation and analgesia through the use of EEG and EMG, respectively, can add valuable information about the state of anesthesia, helping to ensure that the patient does not move or have recall during the surgery.

The most common complication resulting from MEP monitoring is oral trauma secondary to oromandibular contraction during motor tract activation [141, 142]. Oral trauma can take the form of hematoma or laceration of soft tissue within the oral cavity (e.g., tongue, lips, or gingiva), and fracture or avulsion of the teeth. The risk of oral trauma is also heightened during triggered EMG of the facial nerve [143] and presumably the trigeminal, glossopharyngeal, vagus, and hypoglossal nerves. The most common method for mitigating oral trauma is bilateral placement of soft bite blocks between the mandibular and maxillary denta, or between the gingiva in edentulous patients [143–145]. In doing so, the bite blocks often need to be modified to be small enough so as to not obstruct the endoscopic entrance to the nares. Prolonged surgical procedures may heighten the risk of tongue necrosis [146], but it is unclear whether or not proper placement of soft bite blocks may contribute to this risk. It has been suggested that placement of dental guards, in addition to the soft bite blocks, may mitigate the risk of oral trauma [147]. Whenever possible, it is best practice to periodically verify the integrity of the oral cavity as well as confirming that the bite blocks have not become displaced.

## 3. Discussion

The traditional boundaries of the EEA continue to be expanded with advances in instrumentation, optics, and microsurgical techniques. Such expansion requires an intimate knowledge of the anatomy encountered along the ventral skull base and clivus. Even with a profound familiarity of the normal structures encountered, anatomical boundaries and relationships may be distorted by the pathology present, creating difficulty with the identification of landmarks. This is particularly true when faced with anomalous vasculature, which confounds the expected anatomy. A multimodality IONM strategy is required to adequately assess the at risk structures.

When the anterior skull base is accessed, there is considerable concern for the internal carotid arteries and their branches which supply blood to cortical and subcortical structures collectively representing three-fifths of the cerebrum. The most commonly used IONM modalities for monitoring cerebral blood flow are EEG and SSEPs. When one considers the vast array of monitored surgical procedures in which cerebral blood flow is a concern, including cardiovascular, cardiopulmonary, cerebrovascular (e.g., CEA, aneurysm clipping, and vascular malformation), and procedures performed in the interventional radiology suite, one would suspect that EEG is the most widely used vascular monitoring modality. Despite major advantages over other measures such as transcranial Doppler and cerebral oximetry, and numerous studies in which EEG has significantly lowered neurological deficits, shortened postoperative recovery, and reduced hospital costs, there has been little enthusiasm for EEG monitoring in cardiothoracic surgery [37, 148]. The use of EEG in CEA surgery is more widely reported [36, 37], and selective shunting with EEG is safer than routine shunting [33, 149]. In intracranial aneurysm surgery, limited montage EEG monitoring can be used for induced hypotension and neuroprotective burst suppression [37, 150], but more extensive EEG monitoring is impractical, primarily due to the fact that the craniotomy precludes placement of electrodes over the regions at risk for ischemia [151]. This is not the case during surgical procedures using the EEA. Given the risks to the internal carotid arteries and downstream vascular structures in these procedures, the absence of reported EEG monitoring in the literature is unusual, particularly in the context of its demonstrated utility for evaluating cerebral perfusion in cardiothoracic and CEA surgery.

The most likely explanation of the lack of reports specifically addressing EEG monitoring during EEA is that EEG is rather comparable to SSEPs in terms of sensitivity and specificity. Florence and colleagues performed a meta-analysis to compare EEG with SSEPs in terms of their efficacy for monitoring cerebral blood flow [152]. They analyzed outcomes data from different vascular procedures, in which monitoring was performed with either EEG or SSEPs, and found that SSEPs were more sensitive than analog EEG (0.60 versus 0.20); however, their sensitivities were comparable if quantitative EEG was used (0.58). SSEPs and EEG exhibited comparable specificity (0.97 versus 0.95). Other meta-analyses examining EEG and SSEP monitoring during CEA surgery have yielded similar findings [52, 153, 154]. One advantage of extended, multichannel EEG over SSEPs is the ability to localize cortical ischemia beyond SSEP-related watershed regions, giving EEG higher spatial resolution. Also, given the potential for delay in detecting ischemia with SSEPs due to response averaging, EEG presumably has better temporal specificity as well. While the use EEG in EEA has not been specifically reported, it has been advocated for as part of a comprehensive multimodality IONM plan [11, 19, 26].

A major limitation of EEG is that it is purely a measure of cortical activity and, thus, cannot detect subcortical ischemia. For example, if ischemia were isolated to the internal capsule due to reduced flow in the anterior choroidal or lenticulostriate arteries, then the patient may develop hemiparesis in the absence of EEG changes. To compensate for this limitation, SSEP monitoring is introduced to the monitoring plan as a direct measure of somatosensory function. SSEP monitoring during brain surgery has been rather extensively reported. Beyond their sensitivity to cerebral ischemia during a wide range of vascular procedures [51, 155–160], SSEP monitor the integrity of the entire dorsal column-medial lemniscus system, including thalamocortical projections [161]. Furthermore, SSEPs are sensitive to detecting malpositioning of the neck [162, 163] and compression of the limbs which can result in postoperative nerve damage [164–166]. These observations make SSEP monitoring a useful adjunct to EEG monitoring.

The utility of SSEP monitoring during EEA surgery has been investigated in multiple reports by Thirumala and colleagues [11, 12]. In one retrospective review of 999 patients undergoing skull base surgery, the incidence of changes in SSEPs was 20, and there were 5 incidents of new postoperative deficit [11]. In this study there were 2 false negative outcomes of patients who had postoperative deficits (i.e., hemiparesis ± aphasia) in the absence of intraoperative SSEP changes. In a second study of 138 patients undergoing surgery via EEA, SSEP changes were detected in 5 patients, three of which were true positive findings [12]. In both of these studies, intraoperative changes in SSEPs were usually resolved after raising the mean arterial blood pressure (MAP). The authors conclude that SSEP monitoring is a useful adjunct to a comprehensive multimodality IONM plan.

The incidence of false negative findings with SSEP monitoring is not new. Over the years, multiple studies have reported postoperative symptoms ranging from paresis to plegia in the absence of intraoperative SSEP changes [167–171]. For this reason, it is becoming increasing common for tceMEP monitoring to be used during intracranial surgery for direct and indirect (vascular) monitoring of the corticospinal tract [56–66, 172]. Given that SSEPs only directly monitor somatosensory tract function, it makes sense to include tceMEPs into the multimodality IONM plan in an effort to detect evolving motor deficits. When stimulation parameters are kept low to limit the spread of current, tceMEPs are useful in supratentorial tumor resection [57, 58] and aneurysm clipping [56, 60, 61], as well as a multitude of infratentorial/brainstem procedures [62–66]. There are no reports on the use of tceMEPs for the EEA to the skull base. When developing a comprehensive and patient-specific IONM plan in the context of cerebrovascular monitoring, we agree with Thirumala and colleagues [11, 12] in that EEG and SSEPs should be concurrently monitored in all EEA surgeries in which the lesion is in close proximity to critical vascular structures, including the carotid and vertebrobasilar systems. The utility of tceMEPs in these procedures is yet to be conclusively demonstrated; however, with numerous EEG/SSEP false negative reports in the literature, our approach is to monitor tceMEPs on all suprasellar and petroclival lesions.

BAEPs should be added to the multimodality IONM plan when there is risk to the vertebrobasilar system [12, 14]. With the EEA, BAEPs are primarily used to monitor vascular perfusion of the brainstem when the pathology includes the retroclival structures. Intraoperative BAEPs have reliably assessed the integrity and perfusion of both CN VIII and the brainstem during posterior fossa skull base procedures since the late 1970s. Pathology that approximates the basilar artery or the brainstem necessitates the inclusion of this modality into the monitoring plan, as it helps to complete the total clinical picture in regard to potential subcortical ischemia.

The utility of VEPs for intraoperative monitoring of the optic tract is questionable [128]. As the technology for stimulation devices advances, VEPs may play a larger role in monitoring. The TIVA anesthetic protocol helps stabilize the trial-by-trial variability that is present in the VEP responses. Further research is needed to assess if VEPs can be reliably used as an adjunct to monitor procedures where the visual tracts are affected. Presently, there is little evidence from any intracranial procedure that VEPs are reliable predictors of postoperative function. For that reason, they are not recommended as part of a comprehensive IONM plan during EEA to the skull base.

Given the close association with many skull base pathologies to cranial nerves, the utility of cranial nerve monitoring during EEAs is of significant interest. The methodology for eliciting CrN MEPs has been established for recording reliable MEPs from muscles innervated by the facial nerve (CN VII) [70]. Facial CrN MEPs have been successfully employed in a multitude of different surgical procedures, including skull base, brainstem, and posterior fossa [10, 70, 79–81, 173]. Additionally, there is strong evidence in favor of their prognostic value in terms of predicting postoperative facial nerve motor function [79, 81, 173, 174]. Unfortunately, attempts to record CrN MEPs from muscles innervated by other cranial nerves have been met with limited success. The most common motor cranial nerves at risk during the EEA are the oculomotor, trochlear, and abducens nerves [20–22].

To our knowledge, CrN MEPs from the extraocular muscles have not been successfully recorded, probably owing to the fact that the muscles are small and poorly innervated. Additionally, the short onset latency of the response would often be obscured by stimulus artifact. Even if this technique was extrapolated to include these nerves, alarm criteria would need to be established before routine use.

Regarding the trigeminal nerve, there are no published reports of CrN MEPs being recorded from either the masseter or temporalis muscle. We have successfully recorded MEPs from the masseter muscle, but their value remains unclear. First, the origin of the response could be accounted for by volume conduction from the facial muscles. Second, we do not have enough experience with trigeminal MEPs to analyze their prognostic efficacy.

Greater successes have recently been reported for lower cranial nerve monitoring, methods for recording reliable MEPs from the vocal cords were introduced by Deletis et al. [71], and 1 patient with intraoperative unilateral laryngeal nerve injury exhibited immediate reduction in MEPs recorded from the ipsilateral vocalis muscle. Motoyama et al. recently reported their success in recording MEPs from the stylopharyngeus and vocalis muscles in two patients undergoing microvascular decompression for glossopharyngeal neuralgia [175]. Ito et al. successfully recorded MEPs from the vocalis muscles in 15 patients undergoing surgery for skull base or brainstem tumors, and intraoperative changes correlated with postoperative dysphagia [176]. The ability to record reliable MEPs from the upper trapezius muscles for spinal accessory monitoring and the tongue for hypoglossal nerve monitoring have both been reported in isolated patients, but their prognostic value remains unknown [73].

Free-running S-EMG provides real-time feedback of cranial nerve irritation when operating near these sites. Recognition of nerve irritation during a procedure allows for a change in surgical strategy. Given the multitude of different EMG patterns that one may encounter [29], the importance of accurate interpretation cannot be overstated as false positives cause unnecessary concern and have the potential to significantly delay the surgical procedure. Additionally, one should not assume that absence of EMG activity equates with neural integrity [96, 97]. Despite the limited specificity, the utility of S-EMG for monitoring cranial nerve motor function been demonstrated in EEA surgery across the full range cranial nerves [14, 19, 26]. In these studies, S-EMG was useful for identifying the location of a nerve, but was of limited prognostic value.

**Figure 4:**
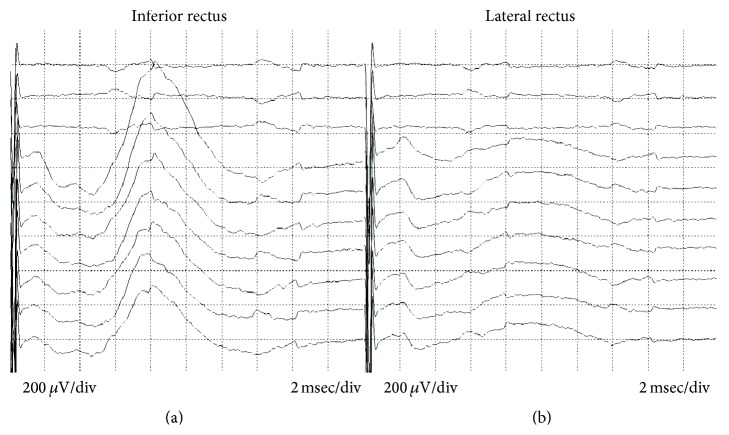
Monopolar stimulation of the left oculomotor nerve at 0.50 mA with subsequent compound muscle action potentials recorded from the left inferior rectus muscle using and intramuscular needle electrode (a). This response is referenced to the contralateral orbicularis oculi muscle. (b) The ipsilateral lateral rectus recording, which was not activated with this stimulation.

The best way to confirm the functional integrity of a motor or mixed sensory/motor cranial nerve is with T-EMG. Whenever possible, the nerve should be stimulated both proximal and distal to the brainstem, on each side of the lesion. In some cases, nerve stimulation is useful to localize cranial nerves and avoid injury. Electrical stimulation of the nerves to the extraocular muscles is feasible and safe (Figure 4) [14, 17, 177–179]. For management of retroclival lesions, the glossopharyngeal nerve (CN IX), vagus (CN X), spinal accessory (CN XI) and hypoglossal (CN XII) nerves can be safely stimulated [10, 180]. Adverse consequences include hypotension and bradycardia (CNs IX and X), and muscle and tendon injuries (CN XI). There are no reported adverse consequences of stimulating CN XII. Maintaining low stimulation intensity with a short (25–100 *μ*sec) duration will help to limit stimulation-induced injury. Electrical stimulation of any motor cranial nerve has the added benefit of evaluating the functional integrity of the nerve, especially in the rare event of nerve transection, which does not necessarily result in spontaneous EMG activity.

Which cranial nerves are monitored will depend on the nature of their involvement of the lesion and the risks associated with the approach (Table 1) [18, 19, 26]. The reliability of T-EMG is largely dependent upon the integrity of the neural elements being tested. Thus, a patient presenting with preoperative neurological deficits confounds the monitoring. Patients with cavernous sinus or suprasellar pathologies will frequently experience preoperative extraocular palsy or visual disturbance. Often, a nerve that is compromised will have an altered threshold, decreased amplitude, increased latency, poor morphology, or no response at all. Marked preoperative clinical neural compromise could create invalid responses, increasing the rates of false positives and negative. In this context, CrN MEPs would be of significant benefit to help establish a neural conduction baseline. Methodological advancements are required to test this hypothesis.

The efficacy of IONM and the modalities chosen by the neurophysiologist to monitor during this approach need to be examined closely, and both case series and case reports are needed to expand the literature base. The monitoring regimen that we present here encompasses a strategy that assesses both cortical and subcortical structures, as well perfusion, and can help to increase the sensitivity and specificity of at risk neural structures during this technically demanding surgical approach.

## 4. Conclusion

IONM has gained widespread acceptance in cranial surgery and has even become standard of care in some settings (e.g., facial nerve monitoring in surgery of the cerebellopontine angle). In this paper, we have presented the modalities employed in varying combinations during endoscopic endonasal skull base surgery. Certainly, not all of these techniques are required for every endoscopic endonasal approach. For example, IONM is unlikely to be beneficial during the resection of standard pituitary adenomas without cavernous sinus invasion. For those lesions that do involve the cavernous sinus and its associated neurovascular structures, or for pathology requiring an EEA, an IONM regimen tailored to the specific approach and the at risk anatomy is utilized (Table 1). In doing so, however, it is necessary to recognize the rationale for each modality as well as its potential limitations.

## Figures and Tables

**Table 1:** Surgical approaches using the endoscopic, endonasal route and recommended IONM modalities based on pathologies commonly encountered via that approach.

Surgical approach	IONM Montage	Common pathology
Transsphenoidal to sella	None	Adenoma, Rathke's cleft cyst
Transsphenoidal, transplanum, transtuberculum to suprasellar region	EEG, SSEPs, MEPs	Meningioma, craniopharyngioma, giant pituitary adenomas
To orbital apex	EEG, SSEPs, MEPs, EMG (CN III, IV, VI)	Hemangioma, meningioma, neoplasm
Transethmoidal, transcribiform to anterior cranial fossa	EEG, SSEPs, MEPs	Meningioma, esthesioneuroblastoma, meningocele
Transclival/transpetrous to posterior fossa	EEG, SSEPs, MEPs, EMG (CN VI, VII)	Chordoma, chondrosarcoma
Transpterygoid	EEG, SSEPs, MEPs, EMG (CN V)	Meningocele, meningoencephalocele, schwannoma
To cavernous sinus	EEG, SSEPs, MEPs, EMG (CN III, IV, VI)	Adenoma, meningioma
Transcondylar/transjugular	EEG, SSEPs, MEPs, EMG (CN IX, X, XI, XII)	Chordoma, chondrosarcoma
